# Public Opinion, Crisis, and Vulnerable Populations: The Case of Title IX and COVID-19

**DOI:** 10.1017/S1743923X20000446

**Published:** 2020-07-09

**Authors:** James N. Druckman, Elizabeth A. Sharrow

**Affiliations:** 1 Northwestern University; 2 University of Massachusetts Amherst

**Keywords:** Title IX, gender, vulnerable populations, COVID-19, coronavirus, sexual harassment, public policy, policy feedback

## Abstract

A central function of democratic institutions is to protect vulnerable populations. The stability and success of these institutions depends, in part, on popular support. Times of crisis can introduce novel dynamics that alter popular support for protective institutions, particularly among those who do not benefit from those protections. We explore this possibility in the context of Title IX's gender equality requirements and infrastructure to address sexual harassment in college sports. We conduct a large survey of college student-athletes to study their attitudes on these issues in response to the COVID-19 pandemic and concomitant financial challenges affecting college sports. We find that male student-athletes and those with sexist attitudes exhibit alarmingly low levels of support for ensuring the maintenance of equality and sexual harassment policy under Title IX during the COVID-19 crisis and eventual recovery. The results accentuate the vulnerability of certain populations during crises and the importance of maintaining strong institutional policy support during such times.

Vulnerable groups—whether due to social, economic, or political forces—often depend on the government for protections. Without regulations and laws, these populations often face discrimination, disenfranchisement, and/or displacement. One prominent example of such a protection is Title IX of the Education Amendments of 1972. This U.S. federal law protects against sex discrimination in educational settings, perhaps exerting its most notable effect by stimulating the massive increase in athletic opportunities for women and girls and, more recently, providing pathways to address sexual assault and harassment. In the abstract, these types of government protections often garner widespread public support, and indeed, when asked about Title IX in general, the public and those in college athletics express strong policy support (Druckman, Rothschild, and Sharrow 2018; Women's Sports Foundation 2017).

Yet periods of societal distress often test the bounds of these protections. This has been the case during the COVID-19 pandemic, which has dramatically impacted the financial well-being of college sports. For example, as of June 2020, 56 colleges had dropped one or more athletic teams. The decisions of athletic leadership could have devastating consequences for Title IX beneficiaries if policy protections go unenforced or unchallenged by target populations. Here, we study stakeholder opinions about Title IX in response to the COVID-19 pandemic (i.e., what aspects of college sports should be protected during the crisis). Using pandemic-specific opinion measures, we find alarmingly low levels of support during the pandemic for protecting equal athletic opportunities *and* sexual harassment infrastructure among male student-athletes—who typically do not directly benefit from Title IX but constitute the majority of student-athletes—and those with high levels of sexist attitudes. The results make clear how crises can alter the opinions of those who are not predisposed to support policy protections and accentuate the importance of strong institutional support to ensure equal opportunities during hard times (see Jabko and Sheingate 2018).

## COVID-19 AND COLLEGE SPORTS

In American college athletics, women's incorporation is ongoing and largely depends on Title IX's implementation. This has led to a dramatic expansion of women's collegiate athletic opportunities and scholarships over nearly five decades. That said, limited enforcement of Title IX at most institutions, where male student-athletes disproportionately benefit from the athletic opportunities and spending, means that women have yet to reach full equality (e.g., Yanus and O'Connor 2016). Furthermore, there is ongoing debate about implementing sexual violence protections in college athletics as a result of several high-profile sexual abuse scandals and recently revised federal policy guidelines. Overall, policy implementation provides women with rights, but women remain among the minority of college athletes, accounting for 43% of current participants, and are beholden to the persistently androcentric world of college sport (Sharrow 2017).

Groups marginalized within or only tenuously incorporated into empowered structures—at work, school, or in public life—are most vulnerable to “crises” because they are susceptible to the retrenchment of rights or benefits (Strolovitch, forthcoming). The COVID-19 crisis is already undermining various measures of gender equality (e.g., Alon et al. 2020), echoing previous findings that women are uniquely vulnerable to the consequences of financial calamity (Blanton, Blanton, and Peksen 2018) and economic catastrophes (Strolovitch 2013).

We theorize that crises have the potential to loosen individuals’ commitments to institutionalized policy protections (Marcus et al. 1995), particularly when personal interest is at stake (Huddy et al. 2002). Title IX's athletic target population, student-athletes, is well suited to test this theory because (a) they have historically exhibited high levels of support for Title IX generally (Druckman, Rothschild, and Sharrow 2018), so shifts in their opinion make for a difficult test of the impacts of crises on rights for marginalized groups, and (b) their future fortunes depend directly on administrative decisions made in response to the COVID-19 crisis. Athlete advocacy has long been key to policy enforcement (Belanger 2016), and thus the impact of crisis on attitudes among this target population may have long-term implications for women's continued incorporation.

We test three preregistered hypotheses:^1^

***H_1_***: Support for Title IX's gender equality requirements and sexual harassment policy will be significantly higher in the abstract than it is in response to the COVID-19 crisis.

***H_2_***: Because women student-athletes are more likely to benefit from Title IX protections, male student-athletes will be less supportive of gender equality requirements and anti-harassment policy protections during the crisis.

***H_3_***: Given the androcentrism which paints sport as a “male domain” (Sharrow 2017), people who harbor sexist beliefs will be less supportive of gender equality requirements and anti-harassment protections during the crisis.

## DATA

To assess our hypotheses, we conducted a survey with a representative sample of 1,925 student-athletes in May and June 2020 (see Appendices 1–3 in the supplementary materials online for details). We initially asked respondents about the extent to which they disagree or agree with the requirements of Title IX (generally) and whether less or more should be done to enforce sexual harassment laws. These items, measured on 7-point scales with higher scores indicating greater support, capture abstract attitudes and were asked early in the survey instrument. To measure opinion in light of the COVID-19 crisis, we included seven items asking whether Title IX's equality of athletic opportunity provision should be relaxed (because of financial strains), whether respondents worry about it being relaxed during the pandemic and the pandemic recovery, whether relaxing compliance requirements would undermine law and equality, and about the relative importance of Title IX vis-à-vis other athletic prerogatives (alpha = .86). We assess opinion toward the protection of infrastructure for addressing sexual harassment during the pandemic using a similar set of seven items (alpha = .84), asked at the end of the survey. Both COVID-19-specific scales range from 1 to 5 with higher scores indicating more support for protecting equality and anti-harassment infrastructure. The midpoint (3) indicates a neutral opinion. Finally, we asked respondents to rate the importance of protecting a host of items, aside from Title IX and anti-harassment infrastructure, in response to COVID-19-related cuts during the recovery (e.g., maintaining current scholarships, coaches, travel resources) on 5-point scales.

We measured the gender of the respondent and sexist attitudes, using the hostile sexism scale (Glick and Fiske 1996) (alpha = .87).^2^ The survey included a host of other variables such as sport, year in school, income, and others, that we use in the analyses (see Appendix 4 for question wording).

## RESULTS

We find middling levels of support for protecting equality and sexual harassment provisions when respondents were asked about each specifically during the COVID-19 pandemic, with respective mean scores on the 5-point scales of 2.98 (SD = .90), and 3.02 (.84).^3^ As predicted by ***H_1_***, these scores are *notably lower* than general support, which has respective means well above the midpoints on the 7-point scales—5.36 (1.73) and 5.52 (1.28), respectively. The abstracted measures are statistically significantly higher than the during-crisis measures (respectively, *z* = 9.89, *p* < .01; *z* = 10.98, *p* < .01).

**Figure 1.**
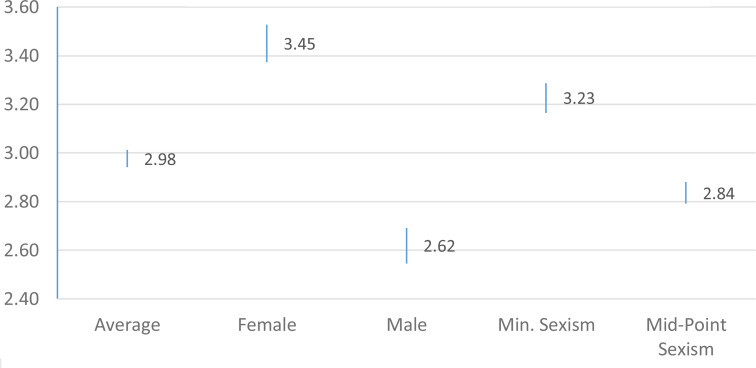
Protecting Gender Equality Requirements In Response to COVID-19 Crisis

**Figure 2.**
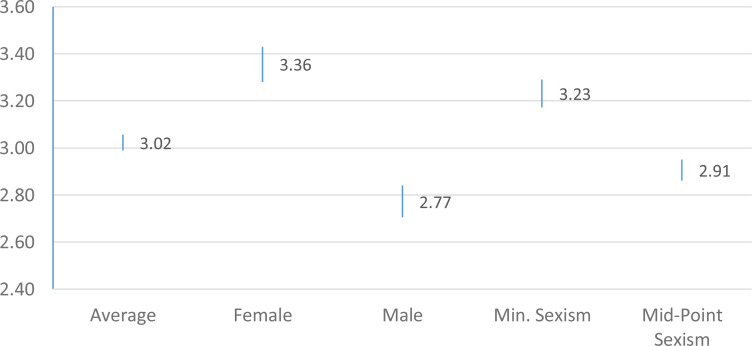
Protecting Sexual Harassment Infrastructure In Response to COVID-19 Crisis

We test our other two hypotheses by regressing the during-COVID-19 equality and harassment variables on gender and sexist attitudes, along with a large host of controls (see Appendix 5 for discussion). The regressions appear in Appendix 5; we plot the predicted values (with 95% confidence intervals) to test our hypotheses in Figures 1 and 2 (using truncated scales).^4^ For the sake of presentation, we present predicted values for sexist attitudes at both the minimum score (1) and the midpoint (4) of the 7-point scale, but the results are similar and significant using other exemplars.^5^

The results offer strong support for ***H_1_*** and ***H_2_***. There are notable differences by gender—specifically, a .83 or 20.75% decrease in support, from women to men, when it comes to protecting equality during COVID-19, and a .59 or a 14.75%, from women to men, decrease in support for safeguarding anti-harassment protections. We see slightly smaller but similar trends among those who exhibit more sexist attitudes. Moreover, men and those with high levels of sexism fall below the midpoints on both scales on average. This exposes that support for these protections is weakened among large sub-groups of college athletes during this crisis period.

**Figure 3.**
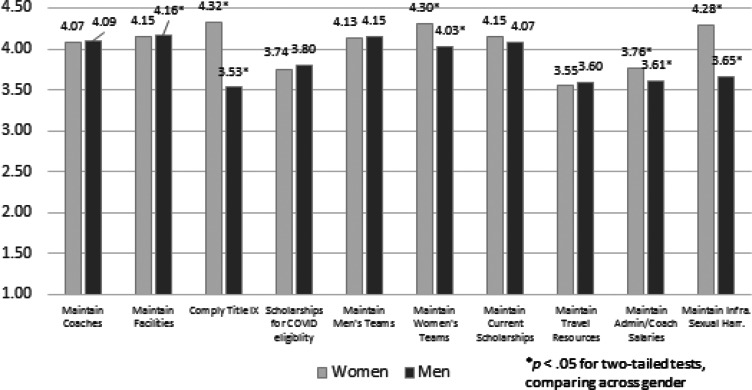
Importance During COVID-19 Recovery

In Figure 3, we plot responses about the relative importance of a host of different financial and policy priorities for athletic departments responding to the COVID-19 pandemic. First, across most items, men and women respond similarly (with the exceptions of attitudes toward cutting women's teams and coaches’ salaries). Second, though, we see substantial differences in protecting Title IX compliance (e.g., equality of athletic opportunity) and maintaining the infrastructure for sexual harassment, both between women and men and compared with other priorities. Here, similar to the foregoing results, we see notable gender disparities that are statistically significant (*p* < .01). For women, these two items are the *highest* scores along with maintaining women teams, while for men they are the two *lowest* scores. Among men, protecting Title IX and the infrastructure to prevent sexual harassment rate no differently than maintaining travel resources (*p* > .29 for men for both travel versus harassment and Title IX, but *p* < .01 for women).

## CONCLUSION

These results underscore how crises—and specifically the COVID-19 pandemic—can have significant consequences for historically marginalized groups. Our findings illustrate the fragile fault lines of support for gendered policies, including Title IX's athletic and anti-harassment protections during such crises, especially among men. These insights echo how the politics of “crises” can often prioritize the desires and recovery of dominant groups while placing the needs of more vulnerable populations at the periphery (e.g., Strolovitch 2013, forthcoming).

Dynamics within the data also highlight the importance of policy knowledge (e.g., Mettler 2018). Despite numerous Title IX investigations by the U.S. Department of Education at colleges around the country (see discussion in Appendix 2), respondents to our survey exhibit very little knowledge about investigations on their campus (among schools with ongoing sexual assault investigations, only 15% were accurately knowledgeable of the investigation). This raises important future research questions about how protections might be valued, during crises or “normal” times, if or when recipient populations accurately comprehend the nature of current enforcement. Further, it remains an open question whether student-athletes react in similar manners during other types of budgetary crises—an important issue for future work given that budgets are inherently tight in college sports despite common perceptions of copious resources.

This study illustrates two lessons for scholars of politics and gender. First, we show why beneficiaries need strong legal protections and enforcement of gender equality policies so that protections are not readily undermined when systems are under stress and/or because of a lack of support from advantaged, majority stakeholders. Second, we illustrate that postcrisis recovery efforts require close oversight on protections for vulnerable groups, irrespective of whether such groups are in a position to advocate on their own behalf. The future for further incorporation of historically marginalized groups will depend on it.

## Footnotes

1. The hypotheses are preregistered at https://aspredicted.org/eq3dr.pdf. We also preregistered an experimental aspect of the study that is outside our purview here.

2. The correlation between the male and sexism variables is .30 (i.e., sexist attitudes are not only present among males).

3. Not surprisingly, the measures are highly correlated at .72.

4. We do so using Clarify.

5. Nineteen percent of our sample is at the minimum sexism score, while the midpoint score is roughly the third quartile. In other words, more than 25% is above it, and hence it is a meaningful number.

## Supplementary material

For supplementary material accompanying this paper visit https://doi.org/10.1017/S1743923X20000446. click here to view supplementary material
