# Supplementary material for: Public Opinion, Crisis, and Vulnerable Populations: The Case of Title IX and COVID-19
Source: Politics & Gender. 2020 Jul 9:1–9. doi: 10.1017/S1743923X20000446 (PMC7573459; doi:10.1017/S1743923X20000446)
Supplement: Supplementary file 1 [file S1743923X20000446sup001.docx]

**Online Appendix 1: Sample Construction**

Our population includes all student-athletes participating in varsity sports at National Collegiate Athletic Association (NCAA) schools.^[[1]](#footnote-1)^ We began by taking a random sample of NCAA schools. At the time of our sampling in the winter of 2020, according to the U.S. Department of Education’s Equity in Athletics Data Analysis (EADA), there were a total of 1,099 colleges and universities in the NCAA.

Of the schools initially selected into our sample, we checked for the availability of publicly-accessible e-mail addresses for student-athletes. If no such e-mails were available, the school was dropped from our sample and replaced with another randomly selected school. If e-mails were available, the school was included in our sample. Our final sample included 57 schools.

We identified individuals for solicitation by identifying all student-athletes listed on the online sports rosters of the institution’s athletic department website. Overall, we ended up with a sample frame of 20,559 individual student-athletes (although see below on e-mail bounce-backs). We acquired e-mails for each of these individuals by accessing the aforementioned rosters and searching for publicly available emails for athletes through each institution’s email search engine.

A fair number of e-mails bounced back to us, presumably due either to the individual no longer being enrolled at the given school, the athletic websites from which we obtained information being out of date, or an incorrectly recorded address. (We ignored auto-responses with the presumption that the e-mail still reached the potential respondent.) Overall, we received 755 bounce-backs; thus our actual sampling frame was 19,804. Our final sample – that is, respondents who completed the entire survey – is 1,925, leading to a response rate of 9.7%. Relative to other targeted samples and other work in this domain – including a COVID-related survey of student-athletes fielded by the NCAA itself during the spring of 2020 for which response was 9% (NCAA 2020) – this response rate is standard.

**Online Appendix 2: Survey Administration**

We administered the survey from May 19, 2020, until June 16, 2020. To each individual for whom we had an e-mail, we sent a personalized invitation inviting him/her to participate in an *anonymous* survey aimed at learning “what student-athletes think about various issues involving college sports” (on personalization, see Druckman and Green 2013). We sent a reminder e-mail roughly one week after the initial invitation and then a second reminder approximately two weeks after the first reminder.

We did not ask individuals to identify their school so as to ensure their anonymity. However, we were interested in knowing whether their school had an open Title IX investigation with the U.S. Department of Education during the prior year. Tracking responses both in light of the athletes’ sponsor institutional status and on questions of athlete awareness of on-going Title IX investigations enables us to observe the extent of policy knowledge among the recipient population (see Mettler 2018). We identified the set of schools that had an open Title IX sports and/or sexual assault investigation as of May 1, 2020, by using the Office for Civil Rights (OCR) of the Department of Education database of open Title IX investigations (<https://www2.ed.gov/about/offices/list/ocr/docs/investigations/open-investigations/index.html>). We used this database to search for all schools in our sample. We next performed Google searches to identify any high-profile Title IX lawsuits that had not been pursued through OCR but which may have permeated the local media/institutional environment during the months preceding our survey. We then grouped the schools into our sample into one of three resulting categories:

1. Schools under no investigations.
2. Schools under an assault investigation only.
3. Schools under both an assault and a sports investigation.

We used distinct survey solicitation links for each type of school so that we could then know the Title IX context the respondent had experienced, while still maintaining anonymity. Among respondents, 36% were from schools with no Title IX investigation, 62% had an assault investigation ongoing and 2% had both. In our analyses, we did check for a direct impact of Title IX investigations (and respondent knowledge thereof) but found no clear effects.

**Online Appendix 3: Sample Demographics**

We weighted our sample based on gender to ensure representativeness. Our weighted sample demographics appear in the below table.

**Appendix Table A: Weighted Sample Demographics**

| **Variable** | **Distribution / Average** |
| --- | --- |
| Gender | Male: 57%; Female: 43% |
| Race (that best describes the respondent) | White: 78%; African-American: 10%; Hispanic/Latino: 4%; Asian/Pacific Islander: 5%; Other: 3% |
| Religion | Protestant: 38%; Catholic: 24%; Non-Christian Religion: 8%; Not Religious: 31% ^A^ |
| Parent With College Degree | 86% |
| High School in the United States | 93% |
| Familial Income | < $30,000: 4%; $30,000-$69,999: 11%; $70,000-$99,999: 18%; $100,000-$200,000: 38%: >$200,000: 30% ^A^ |
| Year in School | First Year: 30%; Sophomore: 26%; Junior: 23%; Senior: 19%; Post-Graduate: 1%^A^ |
| Athletic Scholarship (full or partial) | 36% |
| Academic Scholarship (full or partial) | 38% |
| Coed Team (self-reported) | 9% |
| Athletic Division | Division 1: 48%; Division 2: 10%; Division 3: 42% |
| Control Sports (see appendix 5) | Men’s Basketball: 3%; Football: 12%; Wrestling: 1% |
| Mean Political Ideology (1-7 scale with higher scores indicating more conservative) | 3.55 (std. dev.: 1.51) |
| Mean Racial Conservativism (1 to 7 scale) | 2.55 (std. dev.: 1.18) |
| Mean Hostile Sexism (1-7 scale with higher scores indicating more sexism) | 2.93 (std. dev.: 1.64) |
| Average Percentage Time of Male-Female Student-Athlete Contact | 34% (std. dev.: 15%) |
| Average Percentage Time of African-American/White Student-Athlete Contact | 26% (std. dev.: 17%) |

^A^ This does not sum to 100% due to rounding error.

**Online Appendix 4: Survey Wording**

Which sport(s) did you play at a ***varsity*** level this past academic year? If you played on multiple varsity sports teams, select all teams on which you played. If you did not play due to injury or another reason, select the team(s) with which you affiliate.

| ☐ Acrobatics and Tumbling  ☐ Baseball  ☐ Basketball | ☐ Equestrian  ☐ Fencing  ☐ Field Hockey | ☐ Pistol  ☐ Rifle  ☐ Rodeo | ☐ Squash  ☐ Swimming  ☐ Tennis |  |
| --- | --- | --- | --- | --- |
| ☐ Beach Volleyball  ☐ Bowling  ☐ Cross country  ☐ Diving | ☐ Football  ☐ Golf  ☐ Gymnastics  ☐ Ice Hockey  ☐ Lacrosse  ☐ Lightweight Rowing | ☐ Rowing  ☐ Rugby  ☐ Sailing  ☐ Skiing  ☐ Soccer  ☐ Softball | ☐ Track and Field  ☐ Volleyball  ☐ Water Polo  ☐ Wheelchair Basketball  ☐ Wrestling  ☐ Other  ☐ None |  |

Did you play on a men’s team, a women’s team, or a co-ed team? Check all that apply.

*Men’s Women’s Co-ed*

In which NCAA division did your team(s) compete?

*Division 1 Division 2 Division 3*

What was your year in school this past academic year?

# *First year Sophomore Junior Senior Graduate student N/A*

This past academic year – were you on an **athletic** scholarship, and if so, was it partial or full?

*No athletic scholarship Partial athletic scholarship Full athletic scholarship*

This past academic year – were you on an **academic** scholarship, and if so, was it partial or full?

*No academic scholarship Partial academic scholarship Full academic scholarship*

Did you go to high school in the United States?

__________ __________

*Yes No*

Which of the following best describes your religion?

*Christian Catholic Jewish Muslim Hindu Other Not religious*

What is the highest level of education completed by one of your parents? (Think about the parent who has received the highest level of education.)

*Less than high school High school Some college 4 year college degree Advanced degree*

What is your estimate of your family’s annual household income (before taxes)?

*< $30,000 $30,000 - $69,999 $70,000-$99,999 $100,000-$200,000 >$200,000*

What is your gender?

*Male Female Other*

Which of the following racial or ethnic categories ***best*** describes you (*please check just one on this question*)?

*White Black/African American Hispanic/Latino Asian/Pacific Islander Middle Eastern/ Native Other Northern African American*

Given your knowledge of Title IX, do you disagree or agree with its requirements as applied to college athletics?

*Definitely Somewhat Slightly Neither Slightly Somewhat Definitely*

*disagree disagree disagree disagree nor agree agree agree*

*agree*

Some people think more should be done to enforce sexual harassment laws in college athletics (e.g., within teams, athletic departments). Others think less should be done. What do you think?

*Much Somewhat A little About the A little Somewhat Much more*

*less should be less should be less should be right amount more should be more should be should be*

*done to done to done to is being done done to done to done to*

*enforce enforce enforce enforce enforce enforce enforce*

*sexual harassment sexual harassment sexual harassment sexual harassment sexual harassment sexual harassment sexual harassment*

*laws laws laws laws laws laws laws*

As far as you know, was there a significant controversy (e.g., lawsuit or major debate) regarding Title IX that ***involved athletics*** at your school during this past academic year?

*Not that Yes*

*I know of*

As far as you know, was there a significant controversy (e.g., lawsuit or major debate) regarding Title IX that ***did NOT involve athletics*** at your school during this past academic year?

*Not that Yes*

*I know of*

Of the total time you spent with other student-athletes, what percentage, in the last year, did you spend interacting with each of the below demographic groups. The total cannot exceed 100% but it also need not sum to 100% since we do not list an exhaustive set of demographic descriptions.

White men

Black men

White women

Black women

We hear a lot of talk these days about liberals and conservatives. Here is a scale on which the political views that people might hold are arranged from extremely liberal to extremely conservative. Where would you place yourself on this scale?

*Extremely Liberal Somewhat Moderate; Somewhat Conservative Extremely*

*liberal liberal middle of the conservative conservative*

*road*

*The following statements concern women, men, and their relationships in contemporary society. Please indicate the degree to which you disagree or agree with each statement.*

|  | Definitely disagree | Somewhat disagree | Slightly disagree | Neither disagree nor agree | Slightly agree | Somewhat agree | Definitely agree |
| --- | --- | --- | --- | --- | --- | --- | --- |
| Many women are actually seeking special favors, such as hiring policies that favor them over men, under the guise of asking for “equality.” |  |  |  |  |  |  |  |
| Women are too easily offended. |  |  |  |  |  |  |  |
| Women seek to gain power by getting control over men. |  |  |  |  |  |  |  |
| When women lose to men in a fair competition, they typically complain about being discriminated against. |  |  |  |  |  |  |  |
|  | Definitely disagree | Somewhat disagree | Slightly disagree | Neither disagree nor agree | Slightly agree | Somewhat agree | Definitely agree |

To what extent do you oppose or support affirmative action programs designed to help blacks and other minorities get access to better jobs and education (e.g., a college education)?

*Strongly Moderately Slightly Neither oppose Slightly Moderately Strongly*

*oppose oppose oppose nor support support support support*

*Now we’ll present you with a few statements. After each one, we would like you to tell us how strongly you disagree or agree.*

|  | Definitely disagree | Somewhat disagree | Slightly disagree | Neither disagree nor agree | Slightly agree | Somewhat agree | Definitely agree |
| --- | --- | --- | --- | --- | --- | --- | --- |
| Racial discrimination is no longer a major problem in America. |  |  |  |  |  |  |  |
| Students from disadvantaged social backgrounds should be given preferential treatment in college admissions. |  |  |  |  |  |  |  |
|  | Definitely disagree | Somewhat disagree | Slightly disagree | Neither disagree nor agree | Slightly agree | Somewhat agree | Definitely agree |

*A few more questions about COVID-19. Answer to the best of your ability and do not worry if you are unaware of some of the details about how the NCAA or your school will respond to COVID-19.*

Do you disagree or agree that compliance with Title IX’s equality of athletic opportunity provision should be relaxed during the recovery (to save financial resources)?

a. Strongly disagree that Title IX should be relaxed.

b. Somewhat disagree that Title IX should be relaxed.

c. Neither disagree nor agree that Title IX should be relaxed.

d. Somewhat agree that Title IX should be relaxed.

e. Strongly agree that Title IX should be relaxed.

How worried are you that Title IX compliance requirements (regarding equality of athletic opportunities) will be relaxed during the COVID-19 pandemic?

a. Not worried at all.

b. Slightly worried.

c. Moderately worried.

d. Very worried.

e. Extremely worried.

If Title IX compliance requirements were relaxed, how worried would you be that they would not be restored?

a. Not worried at all.

b. Slightly worried.

c. Moderately worried.

d. Very worried.

e. Extremely worried.

Would you consider the relaxation of Title IX compliance requirements as violating federal law?

a. Definitely not.

b. Probably not.

c. Possibly

d. Probably.

e. Definitely.

Would you consider the relaxation of Title IX compliance requirements as undermining equal opportunity?

a. Definitely not.

b. Probably not.

c. Possibly

d. Probably.

e. Definitely.

Which is a more important priority: ensuring compliance with Title IX or ensuring student-athletes who return for an extra year due to COVID-19 retain the same scholarship support they previously had?

a. Definitely compliance with Title IX.

b. Probably compliance with Title IX.

c. Equally important

d. Probably ensuring the same scholarship support.

e. Definitely ensuring the same scholarship support.

Another area that could come under discussion due to the financial impact of COVID-19 is the investment in infrastructure to strengthen the enforcement of sexual harassment laws in college sports. In light of financial pressures, do you believe that the investment in sexual harassment infrastructure should be decreased, kept the same, or increased during recovery?

a. Definitely decrease investment in infrastructure to enforce sexual harassment laws

b. Probably decrease investment in infrastructure to enforce sexual harassment laws

c. Keep the investment in infrastructure to enforce sexual harassment laws the same

d. Probably increase investment in infrastructure to enforce sexual harassment laws

e. Definitely increase investment in infrastructure to enforce sexual harassment laws

How worried are you that infrastructure for the enforcement of sexual harassment laws in college sports will suffer during the COVID-19 pandemic?

a. Not worried at all.

b. Slightly worried.

c. Moderately worried.

d. Very worried.

e. Extremely worried.

If infrastructure for the enforcement of sexual harassment laws in college sports were relaxed, how worried would you be that it would not be restored?

a. Not worried at all.

b. Slightly worried.

c. Moderately worried.

d. Very worried.

e. Extremely worried.

Would you consider the relaxation of infrastructure for the enforcement of sexual harassment laws in college sports as violating federal law?

a. Definitely not.

b. Probably not.

c. Possibly

d. Probably.

e. Definitely.

Would you consider the relaxation of infrastructure for the enforcement of sexual harassment laws in college sports as undermining equal opportunity?

a. Definitely not.

b. Probably not.

c. Possibly

d. Probably.

e. Definitely.

Which is a more important priority: ensuring the enforcement of sexual harassment laws in college sports or ensuring student-athletes who return for an extra year due to COVID-19 retain the same scholarship support they previously had?

a. Definitely enforcement of sexual harassment laws

b. Probably enforcement of sexual harassment laws

c. Equally important

d. Probably ensuring the same scholarship support

e. Definitely ensuring the same scholarship support

How unimportant or important are each of the below during the COVID-19 recovery:

|  | Very unimportant | Somewhat unimportant | Neither unimportant nor important | Somewhat important | Very important |
| --- | --- | --- | --- | --- | --- |
| Maintaining the same staff/coaches |  |  |  |  |  |
| Maintaining the same level of facilities |  |  |  |  |  |
| Compliance with Title IX |  |  |  |  |  |
| Ensuring student-athletes who return for an extra year due to COVID-19 retain the same scholarship support that had had |  |  |  |  |  |
| Not cutting any men’s teams |  |  |  |  |  |
| Not cutting any women’s teams |  |  |  |  |  |
| Maintaining current scholarship levels. |  |  |  |  |  |
| Maintaining current travel resources for competitions |  |  |  |  |  |
| Maintaining current administrator/coach/staff salaries |  |  |  |  |  |
| Maintaining infrastructure for the enforcement of sexual harassment laws in college sports |  |  |  |  |  |

**Online Appendix 5: Statistical Results**

Appendix Table B includes the regressions used to generate Figures 1 and 2. We included a number of control variables that have been shown to affect attitudes about college sports. We include religion indicators to capture variation in values that may affect gender equity beliefs. We include family income because those with lower incomes may be more supportive of protections during COVID-19 given financial needs. NCAA Division dummies control for the reality that financial concerns around the pandemic impact student-athletes competing in the NCAA Divisions differently (i.e., most of the cancelled teams have been in Division II or III). We include an ideology control because conservatives may generally oppose policy innovation in the domain of sports (Zorn and Gill 2007). We include year in school since Druckman et al. (2014) show year in school impacts attitudes about college sports issues. We control for attendance at a high school in the U.S. because such athletes are likely to be more familiar with debates around Title IX than are international student-athletes. Parental college education (i.e., if any parent has a college degree) is an indicator of student-athlete socio-economic status. We include dummies for whether the respondent has a full or partial athletic or academic scholarship since these individuals may differently value protections during COVID-19 contingent on financial needs. We include a variable indicating self-reported co-ed team membership (primarily track & field/swimming & diving teams) since training in such an environment likely increases gender equality support. We include controls for membership on the football and men’s basketball team given these are the main revenue sports invoked in popular discourse about financials. We include a control for membership on the wrestling team because wrestling has been highly politicized in previous Title IX debates (Sharrow 2020). We include a control for racial identity as a demographic control, and racial conservatism because prior work accentuates the importance of racial attitudes when it comes to opinions about college sports (e.g., Druckman et al. 2016).^[[2]](#footnote-2)^ We include a self-reported measure of contact between male and female athletes because other scholarship indicates it is a relevant predictor of support for gender equity policy; and this same work suggests cross-racial contact conditions college sports attitudes (Druckman and Sharrow 2019).^[[3]](#footnote-3)^ (Indeed, we find here that male student-athletes who interact more with women student-athletes become more supportive.)

The question wordings for these control variables included are in Appendix 4. All the independent variables are scaled from 0 to 1. The N reduces slightly due to item-non-response.

**Appendix Table B: Regressions**

|  | Protect Title IX | Protect Harass |
| --- | --- | --- |
|  |  |  |
| Men’s Basketball Team | -0.004 | -0.052 |
|  | (0.109) | (0.118) |
| Football Team | -0.049 | -0.095 |
|  | (0.066) | (0.068) |
| Wrestling Team | -0.347 | -0.084 |
|  | (0.244) | (0.169) |
| Co-ed Team | 0.004 | 0.034 |
|  | (0.057) | (0.056) |
| NCAA Division 2 | 0.017 | -0.011 |
|  | (0.066) | (0.061) |
| NCAA Division 3 | 0.062 | 0.072 |
|  | (0.049) | (0.047) |
| Year in school | 0.081 | 0.122** |
|  | (0.060) | (0.058) |
| On Athletic Scholarship | -0.050 | -0.061 |
|  | (0.052) | (0.049) |
| On Academic Scholarship | 0.079** | 0.035 |
|  | (0.039) | (0.038) |
| Parental College Education | -0.033 | -0.040 |
|  | (0.054) | (0.057) |
| High School in the US | 0.091 | 0.153** |
|  | (0.064) | (0.063) |
| Income | 0.089 | 0.043 |
|  | (0.074) | (0.078) |
| Catholic | 0.078 | 0.059 |
|  | (0.048) | (0.047) |
| Non-Christian Religion | 0.172** | 0.055 |
|  | (0.072) | (0.065) |
| No Religion | 0.167*** | 0.112** |
|  | (0.048) | (0.048) |
| Ideology (Conservatism) | -0.511*** | -0.594*** |
|  | (0.105) | (0.103) |
| African-American | -0.004 | 0.136* |
|  | (0.074) | (0.077) |
| Hispanic | 0.146 | 0.109 |
|  | (0.099) | (0.102) |
| Asian-American | -0.131* | -0.166*** |
|  | (0.076) | (0.064) |
| Male | -0.833*** | -0.602*** |
|  | (0.078) | (0.080) |
| Racial Conservatism | -0.590*** | -0.717*** |
|  | (0.125) | (0.121) |
| Hostile Sexism | -0.849*** | -0.692*** |
|  | (0.083) | (0.086) |
| Male-Female Contact | 0.743*** | 0.717*** |
|  | (0.181) | (0.197) |
| White-African-American | -0.012 | 0.167 |
| Contact | (0.119) | (0.114) |
| Constant | 3.693*** | 3.596*** |
|  | (0.111) | (0.112) |
|  |  |  |
| Observations | 1,855 | 1,855 |
| R-squared | 0.440 | 0.393 |

Robust standard errors in parentheses

*** p<0.01, ** p<0.05, * p<0.1

**Appendix References**

Druckman, James, Mauro Gilli, Samara Klar, and Joshua Robison. 2014. “The Role of Social Context in Shaping Student-Athlete Opinions.” *PloS ONE* 9 (12): e115159. <https://doi.org/10.1371/journal.pone.0115159>.

Druckman, James N., and Donald Green. 2013. “Mobilizing Group Membership.” *Sage Open* 3 (2): 2158244013492781.

Druckman, James N., Adam J. Howat, and Andrew Rodheim. 2016. “The Influence of Race on Attitudes about College Athletics.” *Sport in Society* 19(7): 1020–39.

Druckman, James N., and Elizabeth Sharrow. 2019. “How Institutions and Social Identity Matter for Social Change.” Paper presented at the Annual Meeting of the American Political Science Association, Washington, D.C. <https://www.ipr.northwestern.edu/our-work/working-papers/2019/wp-19-22.html>

Mettler, Suzanne. 2018. *The Government-Citizen Disconnect*. New York: Russell Sage Foundation.

NCAA, National Collegiate Athletic Association. 2020. *NCAA Student-Athlete COVID-19 Well-Being Survey*. NCAA: Indianapolis, IN. http://www.ncaa.org/about/resources/research/ncaa-student-athlete-covid-19-well-being-study.

Sharrow, Elizabeth. 2020. “A ‘Bridge to Our Daughters’: Title IX Fathers and Policy Development.” In *Stating the Family: New Directions in the Study of American Politics*, eds. Julie Novkov and Carol Nackenoff. Lawrence, KS: University Press of Kansas, 127–63.

Zorn, Christopher, and Jeff Gill. 2007. “The Etiology of Public Support for the Designated Hitter Rule.” *Quarterly Journal of Political Science* 2 (2): 189–203.

1. We thus exclude non-NCAA (e.g., NAIA schools). We also excluded athletes from cheerleading and dance, which do not count in terms of compliance with Title IX or under the Equity in Athletics Disclosure Act (EADA). [↑](#footnote-ref-1)
2. Racial conservativeness is the average of the three relevant items (alpha = 0.6504). [↑](#footnote-ref-2)
3. Men-women contact is the percentage of time men student-athletes report spending with women student-athletes, regardless of race while White-African-American contact is the percentage of time white student athletes report spending with African-American student-athletes regardless of gender. [↑](#footnote-ref-3)
